# Vimentin supports cell polarization by enhancing centrosome function and microtubule acetylation

**DOI:** 10.1098/rsif.2023.0641

**Published:** 2024-06-05

**Authors:** Renita Saldanha, Minh Tri Ho Thanh, Nikhila Krishnan, Heidi Hehnly, Alison Patteson

**Affiliations:** ^1^ Physics Department, Syracuse University, Syracuse, NY, USA; ^2^ BioInspired Institute, Syracuse University, Syracuse, NY, USA; ^3^ Department of Biology, Syracuse University, Syracuse, NY, USA

**Keywords:** cytoskeleton, microtubules, vimentin, cell polarization, centrosome, wound healing

## Abstract

Cell polarity is important for controlling cell shape, motility and cell division processes. Vimentin intermediate filaments are important for cell migration and cell polarization in mesenchymal cells and assembly of vimentin and microtubule networks is dynamically coordinated, but the precise details of how vimentin mediates cell polarity remain unclear. Here, we characterize the effects of vimentin on the structure and function of the centrosome and the stability of microtubule filaments in wild-type and vimentin-null mouse embryonic fibroblasts. We find that vimentin mediates the structure of the pericentriolar material, promotes centrosome-mediated microtubule regrowth and increases the level of stable acetylated microtubules in the cell. Loss of vimentin also impairs centrosome repositioning during cell polarization and migration processes that occur during wound closure. Our results suggest that vimentin modulates centrosome structure and function as well as microtubule network stability, which has important implications for how cells establish proper cell polarization and persistent migration.

## 1. Introduction

Animal cells must be dynamic to move and change shape while also being stable and rigid to generate sustained polarized motion. Central to these features is the cell’s cytoskeleton. The animal cell’s cytoskeleton is comprised of three interconnected filamentous networks: F-actin, microtubules and intermediate filaments (IFs) [1]. These networks coordinate different functional aspects of the cell. Filamentous actin is the cell’s main force-generating machinery, producing protrusive forces at the cell membrane and working with myosin motors to generate cellular contractile forces [2,3]. Microtubules are rigid polarized polymers that span the length of cell and direct cargo transport and organization within the cell [4–6], and IFs form a passive filamentous network that supports and stabilizes the cell [7–10]. Many cellular functions rely on coordinated cross-talk amongst these cytoskeletal networks, though there is much more known about how they work individually than together [11,12].

Vimentin is an IF protein that plays a pivotal role in sustaining the mechanical integrity and motility characteristics of mesenchymal cells. Its significance extends to various biological and pathological processes, notably in wound healing, where its absence in vimentin-null mice markedly impairs this function [13]. Furthermore, a strong association exists between vimentin expression and the enhanced metastasis and aggressive growth of tumours [14–16]. The mechanical behaviour of vimentin and other IF proteins is distinctively different from that of actin or tubulin networks. Vimentin networks are soft at low strain but stiffen and resist breakage when strongly sheared or compressed [17,18]. This property significantly contributes to the viscoelasticity of cells [8,19,20], particularly under significant deformation, safeguarding cells from mechanical damage. Vimentin thus serves as a protective 'cushion', crucial for maintaining nuclear positioning and preventing nuclear damage during scenarios like three-dimensional migration or intense cellular confinement [7,20]. Beyond their mechanical roles, vimentin IFs profoundly influence a range of non-mechanical cellular functions [21,22]. They act as integral regulators of cellular signalling, providing a scaffold that interacts with and modulates various signalling proteins. Recent studies have linked IFs to the integration of signals related to the determination of cell size, cell proliferation, cell fate as well as cell adhesion and motility [23,24].

Several emerging studies point to a mutually reinforcing connection between vimentin IFs and the microtubule network [9,25–27]. Vimentin is an IF protein expressed in mesenchymal cells and highly invasive cancer cells [14,21,28]. Disruption of microtubules collapses the vimentin network [25] and likewise disrupting vimentin alters the microtubule network [27]. Vimentin has been shown to enhance the persistence of the microtubule network by serving as a stable long-standing template for new microtubule growth [9], and recent work has shown in reconstituted *in vitro* systems that vimentin stabilizes microtubules against depolymerization through direct interaction [26]. Together, these works highlight a subtle interplay between microtubules and vimentin that is important for polarized cell migration.

The cell’s centrosome acts as the microtubule-organizing centre of the cell and serves as the main microtubule-nucleating organelle [29]. The centrosome is essential for whole-cell polarization and is typically positioned near the cell nucleus between the nucleus and the leading edge of the cell [30]. Vimentin IFs are also localized around the cell nucleus, forming a dense physical mesh in the perinuclear region of the cell [7,10,21,28]. Experiments with cells on patterned adhesive substrates show loss of vimentin increases variability in centrosome positioning [31]. Cells lacking vimentin also have impaired polarization [32–36], which suggests that vimentin might interact with both microtubules and their organizing centre, the centrosome.

Based on these studies, we hypothesized a functional link between vimentin and the cell’s centrosome in establishing cell polarization and directed migration. Here, we report novel experimental data that addresses the role of vimentin IFs in centrosome structure and microtubule-nucleating function. Our results suggest new mechanisms by which vimentin mediates cell polarization and cross-talks with the cell’s microtubule architecture.

## 2. Material and methods

### 2.1. Cell culture

Wild-type (vim^+/+^), vimentin-null (vim^−/−^) mouse embryonic fibroblasts (mEFs) were kindly provided by J. Ericsson (Abo Akademi University, Turku, Finland). Cells were maintained in Dulbecco’s modified Eagle’s medium including HEPES and sodium pyruvate supplemented with 10% fetal bovine serum, 1% penicillin–streptomycin and non-essential amino acids. Cell cultures were maintained at 37°C with 5% CO_2_.

### 2.2. Immunofluorescence

Cells were fixed for immunofluorescence using methanol for 10 min at −20°C. Cell membranes were permeabilized with 0.05% Triton-X in phosphate-buffered saline (PBS) for 15 min at room temperature and blocked with 1% bovine serum albumin (BSA) for 1 h at room temperature. For vimentin visualization, cells were incubated with primary rabbit anti-vimentin antibody (Abcam) diluted 1 : 200 in 1% BSA in PBS for 1.5 h at room temperature; the secondary antibody anti-rabbit Alexa Fluor 555 (Invitrogen) was used at a dilution of 1 : 1000 in 1% BSA in PBS for 1 h at room temperature. For visualizing microtubules, we used primary alpha-tubulin rat antibody (Bio-rad) diluted 1 : 200 in 1% BSA in PBS for 1.5 h at room temperature and secondary antibody anti-rat Alexa Fluor 647 (Invitrogen) at a dilution of 1 : 1000 in 1% BSA in PBS for 1 h at room temperature. For visualizing acetylated microtubules, cells were incubated with primary acetylated tubulin mouse antibody (Sigma–Aldrich) diluted 1 : 200 in 1% BSA in PBS and incubated for 1.5 h at room temperature; secondary antibody anti-mouse Alexa Fluor 568 (Invitrogen) was used at a dilution of 1 : 1000 in 1% BSA in PBS for 1 h at room temperature. Cells were stained using Hoechst 33 342 (Molecular Probes) for 1 h at room temperature. For visualizing centrosome, cells were incubated with primary Cdk5rap2 rabbit antibody (Bethyl Laboratories), gamma-tubulin rabbit antibody (Sigma–Aldrich), pericentrin rabbit antibody (Abcam), cenexin rabbit antibody (Protein tech) and centrin mouse antibody (EMD Millipore) diluted 1 : 1000 for centrin and 1 : 200 for other centrosomal proteins in 1% BSA in PBS for 1.5 h at room temperature; secondary antibody anti-mouse Alexa Fluor 488 (Invitrogen) for centrin and anti-rabbit Alexa Fluor 488 (Invitrogen) for other centrosomal proteins was used at a dilution of 1 : 1000 in 1% BSA in PBS for 1 h at room temperature. Cells were mounted using Prolong diamond antifade mountant (Life Technologies) for epifluorescence and confocal imaging.

### 2.3. Expansion microscopy

Cells were plated on 22 × 22 mm coverslips until they reached 90% confluence and fixed with ice-cold methanol at –20°C for 10 min followed by the immunofluorescence procedure mentioned above. The cells were stained with antibodies against vimentin and centrin. Expansion microscopy was performed using techniques similar to previously published protocols [37,38]. Briefly, the fixed cells were then incubated at 40°C overnight in incubation solution (30% acrylamide in 1× PBS). Next, 1 ml of freshly prepared gelation solution (20% acrylamide, 7% sodium acrylate, 0.04% bis-acrylamide, 0.5% APS and 0.5% TEMED in 1× PBS) was added per coverslip and allowed to solidify on ice for 20 min, followed by incubation for 20 min at room temperature and additional 1.5 h at 30°C. The solidified gels were then sectioned into 4 mm gel punches using a disposable biopsy punch. The gel punches were then digested with digestion buffer overnight (0.5% Triton-X, 0.03% EDTA, 1 M Tris–HCl, pH 8, 11.7% sodium chloride and 1 U ml^−1^ of proteinase K). Finally, the punches were subjected to a second round of immunofluorescence procedures with antibody concentration increased to twice of the initial concentration and allowed to expand in water overnight at 4°C. The expanded gel punches were then mounted on a 35 mm Mattek dish and imaged using Leica SP8 confocal microscope.

### 2.4. Western blot

Cell lysates were obtained by suspending the cells in lysis buffer (HSEG buffer pH 7.4–40 mM NaCl (Fischer Scientific), 5 mM EDTA (Fischer Scientific), 4% glycerol (Fischer Scientific), 20 mM NaF (Fischer Scientific), 1% TritonX-100 (Fischer Scientific), 1× protease inhibitor; 0.1 mM PMSF). Bio-Rad Protein Assay Kit II (Bio-Rad Laboratories) was used to measure the acetylated tubulin concentration from the post-nuclear supernatant collected from the lysates. Standard western blot procedures were performed. The nitrocellulose membranes were probed with primary acetylated tubulin mouse antibody (Sigma–Aldrich) and primary alpha-tubulin mouse (Sigma–Aldrich) antibody diluted in TBS-Tween20 (ThermoFischer) and incubated overnight at 4°C. The membranes were probed using mouse horseradish peroxidase-conjugated secondary antibody (Invitrogen) for 1 h at room temperature. The protein levels were visualized using Clarity ^TM^ western ECL (Bio-rad Laboratories) substrate and imaged using Bio-Rad ChemiDoc ^TM^ imager.

### 2.5. Microtubule re-nucleation experiments and microtubule analysis

Cells were first treated with 1 µM nocodazole (Sigma–Aldrich) for 30 min at room temperature to disrupt microtubule filaments. To allow for microtubule regrowth, nocodazole was washed out and cells were placed back in cell media. Cells were fixed at 0, 1, 2 and 5 min intervals after washout and stained for microtubules and vimentin. The microtubule regrowth was analysed by tracing the radial region of microtubules stemming from the cell centrosome. Tracing was done manually in FIJI-ImageJ software. A minimum of 75 cells were analysed over 3+ independent experiments per condition. For analysing microtubules after nocodazole treatment, we used the Source Steger’s algorithm in curve trace [39] in FIJI-ImageJ and computed the total microtubule contour length per cell; a minimum of 90 cells were analysed over 3+ independent experiments per condition.

### 2.6. Scratch wound-healing assay

A monolayer of cells was created by culturing 5 × 10^5^ cells in a 35 mm glass bottom Petri dish overnight. Wounds were generated using a 10 µl pipette tip to scratch the monolayer. Following the scratch wound, cells were fixed at different time points (1, 2 and 4 h) post scratching. The monolayers were then stained for centrosome (cdk5rap2), microtubules (alpha-tubulin) and DNA (Hoechst 33342) and imaged using a Nikon epi-fluorescence microscope. Cells within 20 µm of the scratch were analysed for the centrosome position. Centrosome positioning with respect to the wound was analysed using FIJI-ImageJ. A minimum of 70 cells were analysed over 2+ independent experiments per condition.

### 2.7. Imaging

#### 2.7.1. Epi-fluorescence imaging

Epi-fluorescence imaging was performed using a Nikon Eclipse Ti (Nikon Instruments) inverted microscope equipped with an Andor Technologies iXon em+ EMCCD camera (Andor Technologies). Cells were imaged using a Plan Fluor (NA of 1.49) 100× oil immersion objective.

#### 2.7.2. Confocal imaging

Expansion microscopy images of centrosome and vimentin were obtained by using SP8 laser scanning confocal microscope with lightning equipped with HC PL APO 40×/1.10 W CORR CS2 0.65 water objective. Airyscan confocal imaging was used to image microtubules and acetylated microtubules. Images were obtained using Zeiss Airyscan LSM 980 confocal microscope equipped with Plan Apochromat 63× oil immersion objective with 1.4 NA, 8Y multiplex mode and GaAsP detector. Images obtained were processed using Airyscan processing technique from Zeiss Zen 3.2 software. For the scratch assay, zoomed out images at 10× magnification were obtained using spinning disk confocal microscope (Yokogawa CSU-W1) on ab inverted Nikon Ti-E microscope with Perfect focus imaged onto a Andor Zyla CMOS camera.

#### 2.7.3. Statistics

Data are presented as a mean value ± s.e.m. The unpaired Student’s *t*‐test with 95% confidence level was used to determine statistical differences between distributions. Denotations: *p ≤ 0.05; **p ≤ 0.01; ***p ≤ 0.001; ****p ≤ 0.0001; n.s., *p* > 0.05. *N* represents number of independent experiments. *n* represents the number of cells. All the graphs and statistical analysis were done using GraphPad PRISM version 9 (GraphPad Software, Inc.).

## 3. Results

### 3.1. Loss of vimentin perturbs the pericentriolar matrix

**Figure 1.**
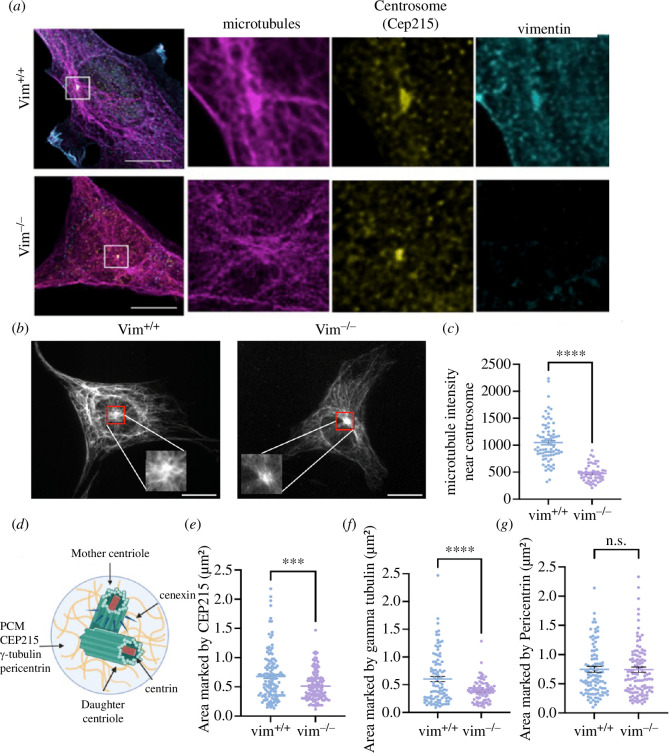
(*a*) Laser scanning confocal images of microtubules, centrosome (CEP215) and vimentin in vim^+/+^ and vim^−/−^ mEF’s. (*b*) Epi-fluorescence microscopy images of microtubules originating from the centrosomes in vim^+/+^ and vim^−/−^ mEF’s . The insets show zoomed-in images at the centrosome. (*c*) Quantification of microtubule intensity near the centrosome (marked as a red box in vim^−/−^ cells) (*d*) Schematic of centrosome structure showing proteins associated with PCM and centrioles. (*e–g*) The projected area of the centrosome marked by CEP215, gamma-tubulin and pericentrin. Denotation: ***p ≤ 0.001; **p ≤ 0.01) *n* = 3, *n* > 90 cells analysed per condition. Scale bar is 20 µm.

To investigate vimentin’s role in cell polarization, we used laser scanning confocal and fluorescence microscopy to examine the centrosome in vim^+/+^ mEFs and vim^−/−^ mEFs (figure 1*a*). To label the centrosome, cells were fixed and stained for the centrosomal protein Cep215 (cdk5rap2), a major pericentriolar material (PCM) protein associated with the organization of microtubules from the centrosome [40,41]. Figure 1*a* shows immunofluorescence images of microtubules, Cep215 and vimentin taken by a confocal laser scanning microscope. In wild-type cells, as expected, there is a strong accumulation of tubulin at the centrosome with long microtubule filaments extending out radially. Interestingly, in vim^−/−^ cells, there is less tubulin localized around the centrosome (figure 1*b*). Further, the centrosome itself (marked by Cep215) is noticeably more condensed and smaller in the vim^−/−^ mEFs compared to vim^+/+^ mEFs. In addition, we observe an accumulation of vimentin that colocalizes with the centrosome in wild-type cells (figure 1*a*).

**Figure 2.**
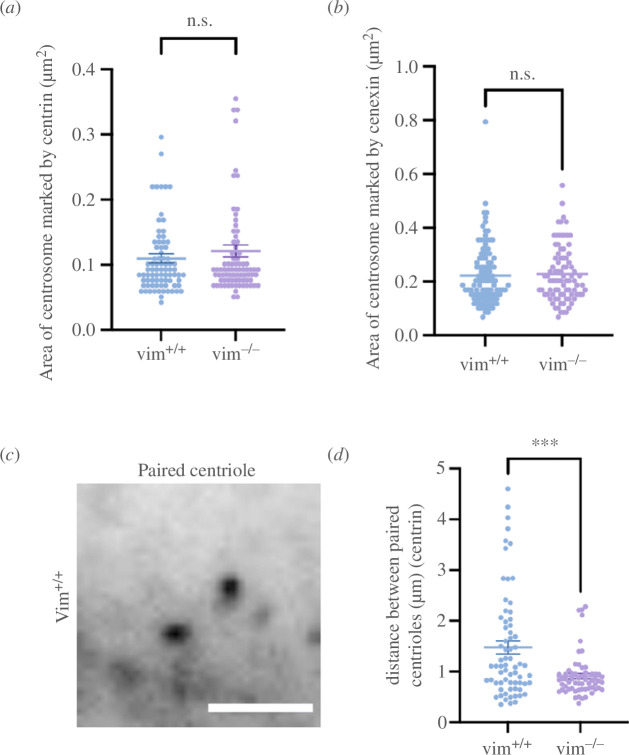
(*a,b*) The projected area of the centrosome marked by centriolar proteins centrin and cenexin. (*c*) Paired centrioles in vim^+/+^ mEF cells. (*d*) Distance between the paired centrioles in vim^+/+^ and vim^−/−^ mEFs. Denotation: ***, *p* ≤ 0.001; ns, *p* > 0.05) *n* = 3, *n* > 90 cells analysed per condition. Scale bar is 10 µm.

Based on these observations, we next sought to quantify centrosome structure in vim^+/+^ and vim^−/−^ mEFs (figures 1 and 2). The centrosome is composed of two centrioles (a mother and daughter centriole) embedded in a dense matrix of PCM proteins (figure 1*d*, schematic). To examine the PCM and centriole structure, we fixed and labelled cells for multiple centrosomal proteins localized in either the PCM (Cep215, gamma-tubulin pericentrin) or centriole protein complexes (cenexin and centrin). We found that the loss of vimentin significantly decreased the mean projected area of the centrosome by 40% as marked by Cep215 (figure 1*e*, *p* ≤ 0.001) and by 40% as marked by gamma-tubulin (figure 1*f*, *p* ≤ 0.001), which indicates that loss of vimentin perturbs the pericentriolar matrix. There was no significant difference in centrosomal area observed by staining for pericentrin (figure 1*g*), but the intensity for cells lacking vimentin is lower compared to wild-type cells (*p* < 0.001, electronic supplementary material, figure S1c), suggesting cells lacking vimentin have less pericentrin protein localized to the centrosome.

Next, we considered the centriole proteins, cenexin and centrin. There was no significant difference in the mean area and intensity of these two centrosome proteins between wild-type and vimentin-null cells (figure 2*a,b*, electronic supplementary material, figure S1*d,e*). However, we did find an impact of vimentin on centriole positioning, namely the distance between centrioles was significantly greater in wild-type cells compared to vimentin-null cells (figure 2*d*). These measurements were obtained for cells with two centrioles. A small fraction of the cells had only one centriole or even less frequently three centrioles, though there was no statistically significant change in centriole number between the two cells (electronic supplementary material, figure S2*b*). From the data in figures 1 and 2, we conclude that loss of vimentin disrupts multiple PCM protein localization as well as centriole position but does not significantly affect the intracellular levels of centriole-associated proteins, cenexin and centrin.

To further examine vimentin and the centrosome structure, we used expansion microscopy to improve the resolution of the images (§2). The centrioles are approximately 200 nm in diameter, just within the spatial limit attainable by light microscopy (200–500 nm). Expansion microscopy overcomes this barrier by physically increasing the size of the specimen by embedding a specimen in a swellable polymer matrix. By swelling the samples, the physical distance between proteins in the cell increases, allowing smaller structures to be resolved by light microscopy.

**Figure 3.**
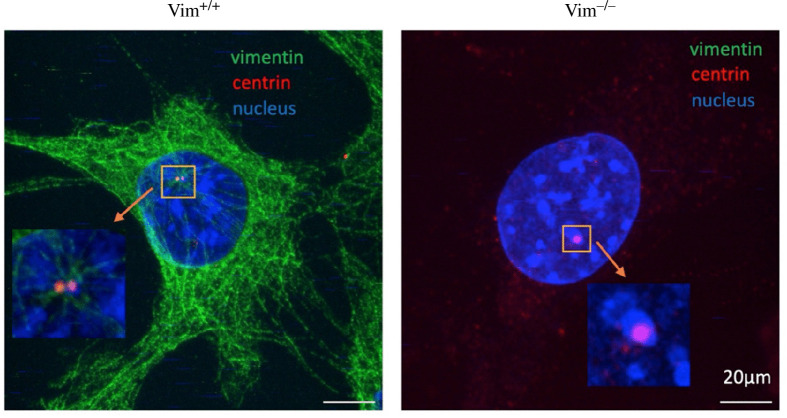
Expansion microscopy images for vim^+/+^ and vim^−/−^ cells stained for nucleus (blue), vimentin (green) and centrosome protein-centrin (red). The inset shows zoomed-in images at the centrosome.

Figure 3 shows representative expansion microscopy images of wild-type and vimentin-null cells labelled with antibodies against vimentin and centrin. In wild-type cells, two distinct centrioles are observable in the expansion microscopy images. An accumulation of vimentin is also observed in the proximity of the centrioles. In contrast, two distinct centrioles are not observable in most of the vimentin-null cells, suggesting the centrioles in vimentin-null cells are much closer together and not spatially resolved even after expansion microscopy. These results further indicate a role of vimentin in maintaining centrosome structure that could be related to supporting the PCM (figure 2) and maintaining the spatial distance between centrioles.

### 3.2. Loss of vimentin disrupts centrosome-mediated microtubule renucleation

**Figure 4.**
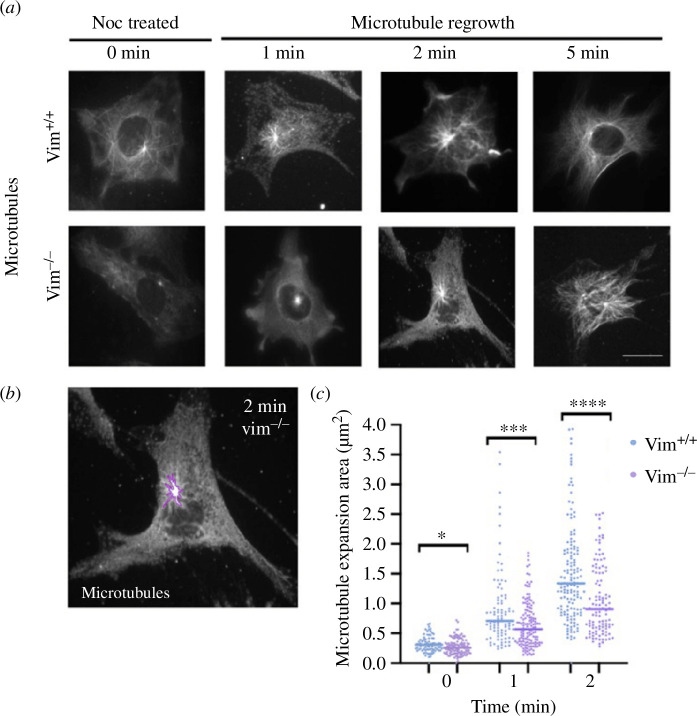
(*a*) Epi-fluorescence microscopy images of microtubules in vim^+/+^ and vim^−/−^ mEFs after nocodazole treatment (0 min) and regrowth at 1, 2 and 5 min after nocodazole washout. (*b*) The area marked to study microtubule renucleation. (*c*) The quantification of microtubule expansion area in vim^+/+^ and vim^−/−^ mEFs for 0,1 and 2 min, respectively. Denotation: *p ≤ 0.05; ***p ≤ 0.001; n.s., *p* > 0.05). *n* ≥ 75 cells analysed per condition, *n* = 3. The scale bar is 20 µm.

Cep215, gamma-tubulin and pericentrin are PCM proteins associated with the organization and nucleation of microtubules from the centrosome [40–43]. Thus, based on our results from figure 1, we hypothesized that loss of vimentin may disrupt the microtubule nucleation function of the centrosome. To quantitatively analyse centrosome-mediated microtubule-nucleating activity, we performed nocodazole-washout microtubule renucleation assays in vim^+/+^ and vim^−/−^ mEFs (§2, figure 4*a*). Microtubules were first disassembled by treating cells with 1 µM nocodazole for 30 min, and then microtubule renucleation from the centrosome was examined at different times after nocodazole washout (0,1, 2 and 5 min). Here, we note our choice to use nocodazole to disrupt the microtubule filaments compared to ice treatment, which is also commonly used. We found that ice was not as effective as the nocodazole conditions used here to remove the microtubule filaments. In particular, we found many cold-stable microtubules in the mEF, consistent with prior literature studies on the mEF cell type [44].

The rate of microtubule renucleation was quantified by tracing the area around the centrosome showing microtubules renucleation (figure 4*b*). At time = 0 min (post-nocodazole washout), the amount of tubulin at the centrosome is slightly lower in vimentin-null cells compared to wild-type cells (12% difference, *p* = 0.04). At 1 min of regrowth, the centrosome in vim^+/+^ showed 28% more microtubule renucleation than in null-cells (*p* < 0.001), and by 2 min, regrowth was 33% greater in wild-type than null-cells (*p* < 0.001). At 5 min, renucleated microtubules span nearly the whole length of cell for both vim^+/+^ and vim^−/−^ mEFs (figure 4*a*). By computing the change in centrosome-nucleated microtubule area over 0–2 min, we find that the area of centrosome-nucleated microtubules grows at a rate 40% higher in vim^+/+^ mEFs (0.64 µm^2^/min) than vim^−/−^ mEFs (0.39 µm^2^/min) (figure 4*c*). While the nocodazole treatment does not completely annihilate the microtubules, the centrosome location is readily apparent, and we quantify microtubule regrowth only from the centrosome. Taken together, the results in figure 4 indicate vimentin may impact centrosome function by enhancing their microtubule-nucleation activity, which could contribute to vimentin’s role in maintaining cell polarization.

### 3.3. Vimentin increases the amount of acetylated microtubules

**Figure 5.**
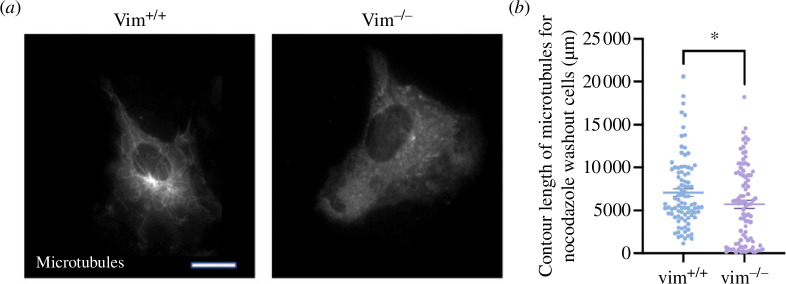
(*a*) Immunofluorescence images of microtubules in nocodazole-treated vim^+/+^ and vim^−/−^ mEFs. Vim^+/+^ mEF’s show the presence of microtubules after 30 min of 1 μM nocodazole treatment (0 min after treatment). (*b*) Quantification of the contour length of the microtubules using the curve fit algorithm from FIJI-ImageJ Denotation: *p ≤ 0.05. *n* > 90 cells analysed per condition for *n* = 3. The scale bar is 20 µm.

On analysing the nocodazole-treated cells (figure 4), we observed the presence of stable microtubule filaments that remained in vim^+/+^ mEFs but not vim^−/−^ mEFs after sustained nocodazole treatment. As shown in figure 5*a*, after nocodazole treatment (*t*=0 min), a number of long microtubule filaments persist in wild-type mEF that are not observed in the null cells. To quantify the amount of remaining microtubules, microtubules were identified using a line/curve detection algorithm (Source Steger’s algorithm, §2) and the total microtubule contour length was computed per cell. As shown in figure 5*b*, wild-type cells had a greater amount of nocodazole-resistant microtubule filaments than vimentin-null cells.

The presence of long-lived nocodazole-resistant microtubules in wild-type mEF indicates a change in microtubule dynamics and stability. This result is consistent with vimentin’s role in supporting cell polarization and enhancing directed motion of cells [34]. Vimentin is known to fortify the microtubule network, serving as a template for new microtubule growth in a feedback mechanism that maintains cell polarity [9] and physically buttresses microtubule filaments against buckling and destabilization [45]. Microtubules dynamically assemble and disassemble with a turnover rate of 3–5 min [46]. Nocodazole disrupts microtubule networks by inhibiting microtubule polymerization; for nocodazole-treatment times greater than the microtubule turnover time, microtubules will depolymerize unless they have been stabilized, for instance by post-translational modifications. Acetylation is a post-translational modification in which an acetyl group is attached to the lysine group (K40) of alpha-tubulin [47]. Acetylation increases the flexibility of microtubules making them more resistant against mechanical forces and breakage [47]. In living cells, microtubules are frequently damaged and subsequently depolymerized; thus, acetylation protects against breaking and increases the stability of microtubules [48].

**Figure 6.**
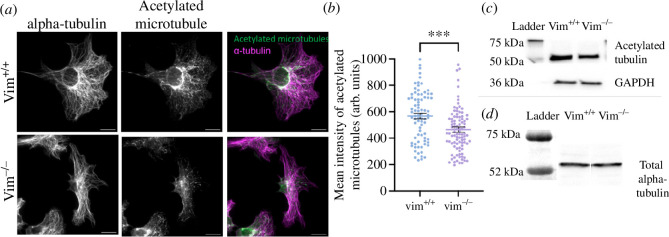
(*a*) Immunofluorescence images of acetylated microtubules for vim^+/+^ and vim^−/−^ mEFs. (*b*) Quantification of mean intensity of acetylated microtubules for vim^+/+^ and vim^−/−^ mEFs. Denotation: ***, *p* ≤ 0.001. *n* ≥ 90 cells analysed per condition, *n* = 3. (*c*) Western blot analysis for acetylated tubulin in vim^+/+^ and vim^−/−^ mEFs with GAPDH as control. (*d*) Western blot analysis for total alpha-tubulin in vim^+/+^ and vim^−/−^ cells. The scale bar is 20 µm.

To investigate changes in microtubule acetylation levels, we next performed immunofluorescence and immunoblotting studies of acetylated tubulin, as shown in figure 6. Figure 6*a* shows immunofluorescence images of acetylated tubulin in vim^+/+^ and vim^−/−^ mEF. In wild-type cells, we observe significant amounts of acetylated tubulin that forms long filaments and a network-like structure in the cell. In contrast, in vimentin-null cells, the acetylated tubulin is much sparser and forms mostly small filamentous squiggles. We note that the mean spread area of vimentin-null cells is larger compared to wild-type cells (electronic supplementary material, figure S3). To further quantify the level of acetylated tubulin, we performed western blot experiments on the wild-type and vimentin-null cells. As shown in figure 6*b*, the mean immunofluorescence intensity of acetylated tubulin is approximately 20% higher in vim^+/+^ cells compared to vim^−/−^ cells (*p* ≤ 0.001). The immunoblotting studies further confirmed that acetylated tubulin is higher in vim^+/+^ cells compared to vim^−/−^ cells whereas the total alpha-tubulin levels in both the cell lines are the same (figure 6*c,d*). Taken together, the results in figures 5 and 6 suggest vimentin positively influences the levels of microtubule acetylation, which enhances microtubule stability.

### 3.4. Vimentin enhances repositioning of the centrosome towards the wound edge

**Figure 7.**
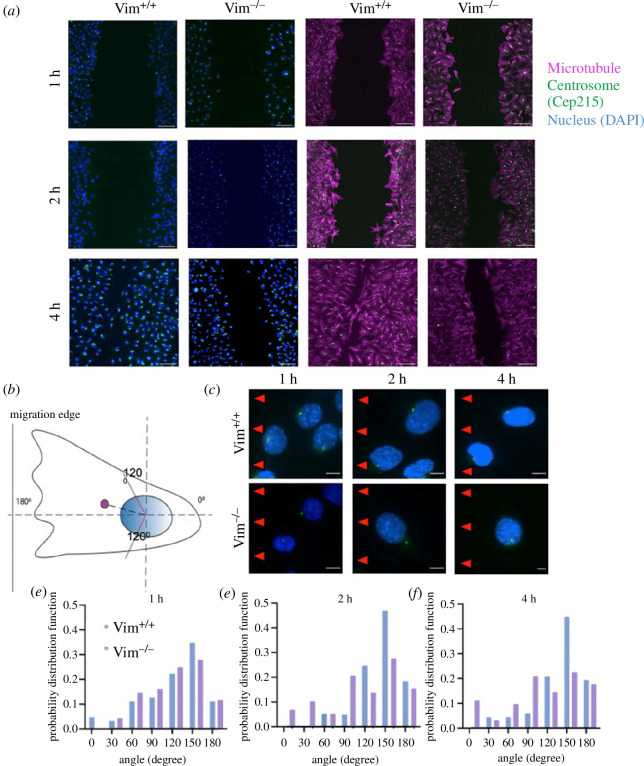
(*a*) Spinning disk confocal images of the nucleus (blue), centrosome protein Cep215 (green) and alpha-tubulin (magenta) for vim^+/+^ and vim^−/−^ mEFs fixed at 1, 2 and 4 h, respectively, after scratch using 10× objective. Scale bar is 100 µm. (*b*) Schematic depicting of the angle made by the centrosome with respect to the centre of the nucleus as the origin axis, the red lines indicate 120° angle (*c*) Zoomed-in images (100× objective) of centrosome (green) and nucleus (blue) of vim^+/+^ and vim^−/−^ cells near the wound edge (20 µm from strach) at 1, 2 and 4 h, respectively (with scratch to the left of the cell). The red arrows indicate the direction of wound. The scale bar is 10 µm (*d–f*) quantification of the centrosome angle for vim^+/+^ and vim^−/−^ cells with respect to the nucleus at 1, 2 and 4 h, respectively. *n* ≥ 70 cells analysed per condition, *n* = 3.

Cells lacking vimentin have been shown to have decreased cell motility [32,49]. As the positioning of the centrosome is important for directional cell polarization and migration, we hypothesized vimentin would also impact the positioning of the centrosome in migrating cells. To study this, we performed an *in vitro* wound healing assay in vim^+/+^ and vim^−/−^ cells. The cells were fixed at 1, 2 and 4 h after making the scratch and stained for centrosomal protein Cep215, α-tubulin and the cell nucleus (DAPI) (figure 7*a*).

On two-dimensional surfaces, the cell centrosome is localized near the nucleus and its positioning between the cell nucleus and the leading edge of the cell tends to demark the polarized directionality of the cell. Upon wounding, fibroblasts at the edge of the wound become characteristically elongated and polarized with their long axis pointing in the direction of the open wound to facilitate wound healing. To investigate the role of vimentin in cell polarization upon wounding, we quantified the ability of the vim^+/+^ and vim^−/−^ mEF to reposition the cell centrosome toward the wound edge. Here, we estimated the cell polarization direction as the vector connecting the centre of the cell nucleus to the centrosome and measured its angular position with respect to the direction of the wound edge, with 180° indicating the centrosome was towards the wound and 0° away from the wound edge (figure 7*b*).

Figure 7*d*–*f* shows the probability distribution function (pdf) of centrosome angle for wild-type and vimentin-null fibroblasts near the wound edge (within 20 µm from scratch) at 1, 2 and 4 h post wounding (§2). As shown in figure 6*d*, at 1 h past wounding, both cell types exhibit a polarization toward the wound edge with a peak at approximately 150°, though there is no statistical difference between the two cell types. No significant differences in the centrosome positioning were observed for regions far away from the wound (electronic supplementary material, figure S4). At 2 h past wounding, the peak at 150° increases from a pdf value of 0.349–0.469 for wild-type cells but remains approximately the same for vimentin-null cells. At 4 h, a peak at 150° remains for wild-type cells, whereas the pdf is levelling out in vimentin-null cells. To further quantify the centrosome positioning, we calculated the percentage of cells with centrosome angles above and below a 120° threshold, with centrosome angle above 120° indicating a polarized direction toward the wound edge (table 1*a*). At 2 and 4 h post wounding, there is a statistically significant increase in polarized cells in wild-type mEF compared to vimentin-null mEF. At 2 h, approximately 65% of the vim^+/+^ cells are polarized, whereas, for the vim^−/−^ cells, only 40% of cells showed centrosomes aligned towards the wound (*p*=0.0018,**). At 4 h, again we see similar results, with 64% vim^+/+^ cells having their centrosomes positioned towards the wound edge, compared to only 40% vim^−/−^ cells (*p* = 0.0007,***). Taken together, the data in figure 7 indicate that vimentin enhances cell polarization during wounding healing and supports the repositioning of the centrosome towards the wound edge for directional cell migration to close the wound.

**Table 1.** Percentage of cells with centrosome angle above 120°. Percentage of vim^+/+^ and vim^−/−^ cells having centrosome angles above 120° and *p*-values using chi-square test for 1, 2 and 4 h, respectively.

time (h)	vim^+/+^	vim^−/−^	*p*-value using chi-square test
1	54	60	0.3915, n.s.
2	65	43	0.0018**
4	64	40	0.0007***

## 4. Discussion

Coordinated polarized cell migration requires an organized effort of three cytoskeletal networks, F-actin, microtubules and vimentin, but the details of how these distinct networks interact to enable cell motility remain largely unclear. Our results here suggest that vimentin networks are important for centrosome function and stabilize microtubule dynamics. In the presence of vimentin, we found that the size of centrosome is amplified, and centrosome-mediated microtubule nucleation is enhanced. The presence of vimentin also increases levels of microtubule acetylation, boosting microtubule stability in the cell. Further, during wound-healing experiments, vimentin increases repositioning of the cell centrosome toward the wound edge, supporting polarized cell migration to close the wound. From these observations, we propose that vimentin can modulate centrosome structure and function as well as microtubule network stability to enhance cell polarization.

One of the most pronounced phenotypes of vimentin-null mice is impaired wound healing [13,50]. *In vitro* wound-healing assays have shown that disrupting vimentin blocks directional migration and delays wound healing in several different cell types, including mEF [49], primary astrocytes [49], retinal pigment epithelial cells [9] and polyploidal giant cancer cells [51]. This loss of directional wound healing can be attributed to a combination of factors, including reduced cell stiffness [19,20], altered nuclear positioning [10], altered cellular traction forces [33,36,52] and impaired directional migration [9,32]. Our results shed new light on vimentin’s role in wound healing via centrosome positioning and tubulin acetylation that are necessary for the proper polarization and directional motion for cells to heal wounds.

Recent work is revealing intricate, complex pathways at the signalling cross-roads among vimentin, microtubules and F-actin in coordinating cell dynamics. Prior work by Jiu *et al.* indicated that vimentin is antagonistic to actin stress fibre assembly through the microtubule-associated guanine nucleotide exchange factor GEF-H1 that activates RhoA [53]. FRAP studies showed loss of vimentin increased GEF-H1 dynamics and increased GEF-H1 phosphorylation on Ser886, which was attributed to increased GEF-H1 activity in the absence of vimentin by using a phosphomimetic mutant protein [53]. Our results here suggest a possible parallel pathway for increased GEF-H1 activity in vimentin-deficient cells through altered microtubule acetylation (figure 5): we speculate that the increased levels of actin stress fibre assembly and cellular traction stresses in vimentin-deficient cells, which have been observed before, arises from the loss of stable acetylated microtubules, which releases GEF-H1 and increases the soluble pool of GEF-H1 that activates RhoA. On the other hand, recent work on microtubule acetylation by Seetharaman *et al.* would predict the reverse effect. Seetharaman *et al.* found that microtubule acetylation can promote the release of GEF-H1 from microtubules to activate RhoA and actomyosin contractility [54]. This indicates a mechanism by which GEF-H1 activity increases with the level of acetylated microtubules, which here is greater in wild-type than vimentin-null mEF and would thus predict an increase in actin stress fibre assembly and contractility in wild-type cells, which we note has been observed in some contexts, such as cells plated on soft substrates or in soft gels compared to on rigid substrates [45,49,55]. These two different expected effects of increased microtubule acetylation in vimentin-expressing cells on RhoA activity through GEF-H1 suggest at least two distinct biochemical signals that engage vimentin in regulating GEF-H1 activity and actin stress fibre formation. Of note, a recent study showed a link between centrosome amplification, an increased number of centrosomes commonly found in cancer cells, with increased tubulin acetylation levels [56]. Further, the polarized distribution of acetylated tubulin played a role in positioning the cell centrosome, distancing it away from the cell nucleus and organizing cell polarization.

Our results suggesting vimentin IFs impact centrosome size and function might be surprising, given that the primary function of the cell centrosome is organizing microtubules. Given the interdependent organization of vimentin and microtubules, early work in the IF field examined a possible link between vimentin and the centrosome [57,58]. Results have been mixed. One of the first studies using electron microscopy in 1985 found no direct interaction between centrioles and vimentin in HeLa cells [57], whereas later in 1995, an association between vimentin and PCM was found in SW-13 cells stably transfected with low levels of vimentin [58]. Our results indicate a possible functional role of vimentin in the microtubule-nucleating capacities of the centrosome. Our results suggest that vimentin impacts the size of the pericentriolar material of the cell centrosome, though it is not yet clear whether the change in size is due to altered spatial distribution of centrosomal protein or a change in the amount of PCM protein per centrosome, which are both possible structural changes for the centrosome [59]. While there are relatively few studies on the association of vimentin and the centrosome, we note that vimentin was identified in a 2003 proteomic characterization of the human centrosome by protein correlation profiling [60]. We note that one limitation of this study is that we did not measure intracellular levels of the pericentriolar proteins. Prior studies have indicated that the PCM behaves as a liquid condensate and the amount of pericentriolar material depends on the overall protein levels inside the cell [61,62]. It would be interesting in future studies to consider the effects of vimentin on transcriptional, translation or degradation levels of such proteins. Taken together, these works highlight a need to re-examine the link between vimentin, centrosome and its implications for whole cell organization and polarity.

Finally, our results here suggest new mechanisms by which vimentin coordinates polarized cell migration by regulating centrosome function and microtubule stability. Our recent work has shown loss of vimentin leads to abnormally persistent cell migration through confining microfluidic channels and that the presence of vimentin is needed for the ability of cells to stop and turn around [33,34]. In confining three-dimensional spaces, such as tissue, tight spaces add extra constraints and restrictions to motion of the cytoskeleton. In many (though not all) three-dimensional settings, the centrosome trails the cell nucleus, defining the tailing direction of the cell, and for the cell to change direction, the centrosome must reposition around the nucleus to a new trailing side of the cell [63]. We propose that by promoting centrosome activity vimentin boosts the ability of the centrosome to dynamically organize and reorganize microtubules and thus, the capacity of the cell to coordinate polarized, directional motion. The microtubule network stabilizing effect of vimentin may further allow the cell to build an internal compass that competes against externally imposed constraints.

## 5. Conclusion

Taken together, our results demonstrate a new role for VIFs in maintaining centrosome size and microtubule network stability. Our data provide evidence that VIFs are involved in regulating the microtubule-nucleating function of the cell centrosome and the expression levels of acetylated tubulin. Further, during wound-healing experiments, vimentin increases repositioning of the cell centrosome toward the wound edge, supporting polarized cell migration to close open wounds. Our results provide new insight into the coordinated cross-talk between vimentin and the microtubule networks in cell polarization.

## Data Availability

All data can be accessed from the Dryad digital repository [64]. Electronic supplementary material is available online [65].
